# Chinese patent medicine Bailing capsule for treating lupus nephritis: A protocol for systematic review and meta-analysis

**DOI:** 10.1097/MD.0000000000017041

**Published:** 2019-09-13

**Authors:** Hui-Jie Ren, Ya-Lei Sun, Bin Yuan

**Affiliations:** Department of Pediatrics, Affiliated Hospital of Nanjing University of Chinese Medicine, Nanjing, Jiangsu, China.

**Keywords:** Bailing capsule, lupus nephritis, meta-analysis, protocol, randomized controlled trials

## Abstract

**Background:**

Treatment of lupus nephritis (LN) remains challenging. LN remains the primary source of morbidity and mortality in patients with systemic lupus erythematosus. Chinese patent medicine Bailing capsule is commonly used for the treatment of LN. However, there is no consensus on the difference in clinical efficacy compared with routine treatment. Therefore, we plan to perform a systematic review and meta-analysis to review the clinical efficacy and safety of Bailing capsule for LN.

**Methods:**

Cochrane Central Register of Controlled Trials, PubMed, EMBASE, Chinese National Knowledge Infrastructure, Chinese Biomedical Database, Wanfang database, and VIP information database will be searched from their inception until January 2019. The meta-analysis will be conducted with Review Manager 5.3 software to systematically review the clinical efficacy and safety of Bailing capsule for LN. The primary outcome will include clinical effective rate, and the secondary outcomes will include Systemic Lupus Erythematosus Disease Activity Index, serum creatinine, 24-hour urine protein quantity, complement 3, and adverse effects.

**Results:**

This analysis will provide useful information about clinical efficacy and safety of Bailing capsule for LN.

**Conclusions:**

Our study will generate strong evidence of Bailing capsule for patients with LN and provide suggestions for clinical practice.

## Abstract 2

Supplemental Digital Content is available in the text

## 1 Introduction

Systemic lupus erythematosus (SLE) is a very complex chronic inflammatory disease. Its clinical features involving severe and persistent inflammation that damages several organs, including the skin, kidney, joint, and brain.^[1]^ The prevalence is an estimated 20 to 200 cases per 100,000 persons.^[2]^ Although the disease affects both males and females, women of childbearing age are diagnosed 9 times more than men.^[2,3]^ Patients with SLE are more likely to have lupus nephritis (LN). It has been reported that more than half of SLE patients suffer from LN,^[4,5]^ which is also the leading cause of mortality by SLE.^[6,7]^Most patients develop LN during the prime of their lives.^[8]^

To date, treatment of patients with LN is primarily immunosuppressant. However, related research showed immunosuppressants used in treatment put patients at a higher risk of infections.^[9]^ For instance, a study shows kidney biopsies after therapy with corticosteroids and cyclophosphamide show an increase in chronic damage.^[10,11]^ Meanwhile, unwanted side effect is of concern. The application of immunosuppressants came with a series of adverse events. For example, ischemic and valvular heart disease, osteoporosis, and premature menopause.^[12–14]^ These detrimentally affect their livelihoods and families, affecting the whole society. The last years has seen a tremendous amount of clinical trials from well-conducted studies on how to best treat LN by achieving profitable outcomes with the least amount of therapy medicine toxicities. However, the disease burden of LN remains large, particularly among young women.^[15]^ Limitations of a better understanding of the pathogenesis of the disease led to significant therapeutic advances that have not occurred. Therefore, new regimens are still actively being sought.

In consideration of limitations, the application of Chinese patent medicine Bailing capsule could be promoted. Chinese patent medicine Bailing capsule is a good choice for the treatment of LN. In recent years, randomized controlled trials (RCTs) on the treatment of LN with Bailing capsule have been reported. However, compared with routine treatment, there is no consensus on the difference in clinical efficacy. Therefore, we plan to conduct this systematic review and meta-analysis to systematically review the clinical efficacy and safety of Bailing capsule for LN. This analysis is expected to obtain meaningful conclusions and provide a high level of evidence-based medicine evidence for the Bailing capsule treatment of LN.

## 2 Methods

### 2.1 Study registration

The study protocol has been registered on PROSPERO CRD 42019126587.

### 2.2 Inclusion criteria

#### 2.2.1 Types of studies

RCTs using Bailing capsule to treat LN regardless of blinding, allocation concealment.

#### 2.2.2 Types of patients

The study will include patients who were diagnosed as LN according to “systemic lupus erythematosus diagnosis and treatment guidelines.”^[16]^ There were no restrictions on gender, age, course of disease, and course of treatment.

#### 2.2.3 Types of interventions

The treatment group received Bailing capsule, and the control group received routine treatment (e.g., prednisone, tacrolimus, and so forth). On the basis of these, patients could receive relevant therapy if they had complications during therapeutic process.

#### 2.2.4 Types of outcome

The primary outcome for this meta-analysis is clinical effective rate. The efficacy criteria will be predominantly based on reduction of clinical symptoms and the recovery of biochemical indicators. The rate will be calculated by this formula: (number of remarkable recovery patients + number of clinical cure patients) / total number × 100%. Besides, Systemic Lupus Erythematosus Disease Activity Index (SLEDAI), serum creatinine, 24-hour urine protein quantity, complement 3, and adverse effects were also evaluated.

### 2.3 Search strategy

We will perform the systematic search in both English and Chinese database from their inception to January 2019: Cochrane Central Register of Controlled Trials (CENTRAL), PubMed, EMBASE, Chinese National Knowledge Infrastructure, Chinese Biomedical Database, Wanfang database, and VIP information database. There are no language restrictions; meanwhile, the medical subject headings (MeSH) and free text words will be applied. The search strategy will be formed by 3 items: health condition (LN), Bailing capsule, and study type (RCTs). Detailed strategies are shown in Additional file 1. In addition, reference lists of identified papers will also be checked.

### 2.4 Study selection and data extraction

**Figure 1:**
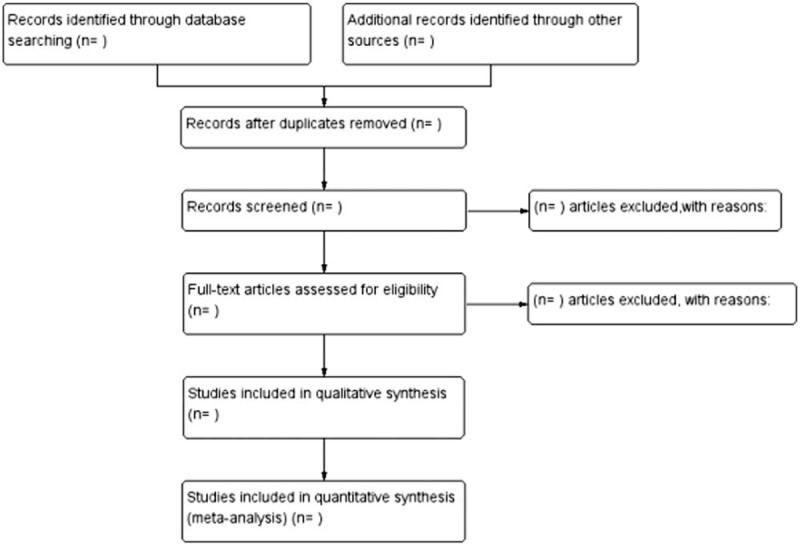
Flow diagram of the study search.

Retrieved papers will be managed by NoteExpress 3.0 software (Beijing AiQingHaiYueZhi Technology Co., Ltd. Beijing, China). Two review authors independently read the tittles and abstracts to exclude wrong literature (e.g., irrelevant, review, and animal experiment). The rest literature read full text to sort out the eligible. The data extraction items include: author names, publication date; age, gender, number of patients, and course of disease; interventions, course of treatment; and outcomes. Any disagreement will be resolved by discussion with a third review author. The selection process will be provided in Figure 1.

### 2.5 Risk of bias assessment

The methodological quality of the eligible studies will be evaluated by 2 review authors according to the Cochrane Handbook for Systematic Reviews of Interventions.^[17]^ The details include: random sequence generation, allocation concealment, blinding of participants and personnel, blinding of outcome assessors, incomplete outcome data, selective reporting, and other bias. Each entry will be assessed as “low risk” or “unclear risk” or “high risk.” Any disagreement will be resolved by discussion with a third review author.

### 2.6 Statistical analysis

Cochrane Collaboration's Review Manager 5.3 software (Copenhagen) will be used to perform the meta-analysis. For dichotomous variable, the results will be calculated as odds ratios. For continuous variable, mean differences will be used. Both of them corresponding 95% confidence interval will also be calculated. Heterogeneity will be assessed using the *I*^2^. *I*^2^ < 50% indicate that the studies have homogeneity, so fixed effects model will be used, otherwise the random effects model will employed for analysis. Subgroup analysis for outcomes will be performed based on prespecified effect modifiers as follows: study quality, sample size, age, gender, treatment duration, etc. If the data are not available for quantitative analysis, we will report result by qualitative description. If adequate trials are included in the study (>10 trials), funnel plot and Egger test will be performed to detect publication bias.

### 2.7 Quality of evidence

We will use a grade framework to assess the quality of evidence attributed to each outcome, with quality-assessment domains including study limitations, imprecision, inconsistency, indirectness, and publication bias.

## 3 Discussion

In recent years, the RCTs of Chinese patent medicine Bailing capsule for the treatment of LN have gradually increased. Numerous literatures have suggested that the application of Bailing capsule holds a significant position for LN. However, the difference in clinical efficacy compared with routine treatment is uncertain. This meta-analysis will be the first review to review the effectiveness of Bailing capsule for the treatment of LN. We hope that the results of our study will provide the clinical recommendation for patients with LN, and promote evidence-based for clinical Chinese patent medicine.

## Author contributions

**Conceptualization:** Ya-Lei Sun.

**Formal analysis:** Hui-Jie Ren.

**Software:** Hui-Jie Ren.

**Writing – original draft:** Ya-Lei Sun, Bin Yuan.

**Writing – review & editing:** Bin Yuan.

Bin Yuan orcid: 0000-0002-6071-8925.

## Supplementary Material

Supplemental Digital Content
