# Supplementary material for: Chinese patent medicine Bailing capsule for treating lupus nephritis: A protocol for systematic review and meta-analysis
Source: Medicine (Baltimore). 2019 Sep 13;98(37):e17041. doi: 10.1097/MD.0000000000017041 (PMC6750295; doi:10.1097/MD.0000000000017041)
Supplement: Supplemental Digital Content [file medi-98-e17041-s001.doc]

Search Strategy

#1 "Lupus Nephritis"[Mesh]

#2 "Lupus Glomerulonephritis"[Title/Abstract]

#3 "Nephritis, Lupus"[Title/Abstract]

#4 "Nephritides, Lupus"[Title/Abstract]

#5 "Glomerulonephritis, Lupus"[Title/Abstract]

#6 "Glomerulonephritides, Lupus"[Title/Abstract]

#7 "Lupus Glomerulonephritides"[Title/Abstract]

#8 #1 OR #2 OR #3 OR #4 OR #5 OR #6 OR #7

#9 "Medicine,Chinese Traditional"[Mesh]

#10 "Chinese Medicine"[Title/Abstract]

#11 "zhongyi"[Title/Abstract]

#12 "zhongyao"[Title/Abstract]

#13 #9 OR #10 OR #11 OR #12

# #14 "Bailing capsules"[Mesh]

#15 "Bailing capsule"[Title/Abstract]

#16 "Chinese patent medicine"[Title/Abstract]

#17 "Chinese patent medicine bailing capsule"[Title/Abstract]

#18 #14 OR #15 OR #16 OR #17

#19 randomized controlled trial[Publication Type]

#20 controlled clinical trial[Publication Type]

#21 randomized[Title/Abstract]

#22 randomly[Title/Abstract]

#23 placebo[Title/Abstract]

#24 trial[Title/Abstract]

#25 groups[Title/Abstract]

#26 drug therapy[MeSH Terms]

#27 drug therapy[Subheading]

#28 #19 OR #20 OR #21 OR #22 OR #23 OR #24 OR #25 OR #26 OR #27

#29 #8 AND #13 AND #18 AND #28
